# Does fibrosis have an impact on survival of patients with hepatocellular carcinoma: evidence from the SEER database?

**DOI:** 10.1186/s12885-018-4996-z

**Published:** 2018-11-16

**Authors:** Hui Liu, Dong Cen, Yunxian Yu, Yanting Wang, Xiao Liang, Hui Lin, Xiujun Cai

**Affiliations:** 1 Zhejiang Provincial Key Laboratory of Laparoscopic Technology, Sir Run Run Shaw Hospital, School of Medicine, Zhejiang University, Hangzhou, China; 2 Department of General Surgery, Sir Run Run Shaw Hospital, School of Medicine, Zhejiang University, Hangzhou, China; 3 Department of Epidemiology and Health Statistics, School of Medicine, Zhejiang University, Hangzhou, China; 4 Department of Internal Medicine, John H Stroger Hospital of Cook County, Chicago, IL USA

**Keywords:** Hepatocellular carcinoma, Liver fibrosis, Survival, Decision curve analysis, SEER database

## Abstract

**Background:**

Liver fibrosis is involved in hepatocellular carcinoma (HCC), but its effect on the survival of patients with HCC remains controversial. This study aims to explore whether the severity of liver fibrosis has an impact on HCC overall survival (OS) and disease-specific survival (DSS) in Surveilance, Epidemiology, and End-Results (SEER) database.

**Methods:**

A total of 11,783 HCC patients diagnosed between 2004 and 2014 from SEER database were enrolled. Cox proportional hazard regression models were used to estimate crude and adjusted hazard ratios (HRs) with 95% confidence intervals (CIs) for fibrosis group associated with survival. Decision curve analysis (DCA) was also performed to compare the effect of fibrosis with other clinicopathological characteristics for survival outcome.

**Results:**

Patients with high fibrosis score (5–6) had a greater proportion than those with low fibrosis score (0–4) (80.3% vs. 19.7%). Fibrosis score was an independent prognostic factor for OS (HR = 1.09, 95%CI: 1.02–1.16), but not for DSS (HR = 1.05, 95%CI: 0.98–1.13) by multivariate Cox proportional hazard models. Additionally, there was no significant effect of liver fibrosis on OS and DSS with stratification of TNM stage and therapy. Findings of DCA showed that fibrosis was less associated with survival outcome in comparison with other tumor characteristics.

**Conclusions:**

The effect of fibrosis on HCC survival was less important than that of other clinicopathological characteristics (like TNM stage or tumor size).

## Background

Liver cancer is the fifth and ninth most prevalent malignant cancer in men and women worldwide, respectively [1]. Every year approximately 745,500 patients die of the disease, making it the second leading cause of cancer-related death among men in the world [1]. Hepatocellular carcinoma (HCC) is the most common primary malignancy of the liver, and accounts for 65% of all cases of liver cancers in the United States surveillance, epidemiology, and end results (SEER) database program [2] and its annual incidence is increasing worldwide [3, 4]. Surgical resection, ablation, chemotherapy and liver transplantation are the main curative treatments [5, 6], but the management of HCC remains disappointing because of its high frequency of metastasis and recurrence [3].

Liver fibrosis, a kind of liver tissue scar reaction involved in the chronic liver injury, is the process from the chronic liver disease to cirrhosis [7]. Moreover, in majority of cases, HCC develops in the setting of bridging fibrosis, a progressive process in which chronic inflammation and hepatocellular regeneration result in the production of reactive oxygen species, chromosomal mutations and eventually, malignant transformation of proliferating hepatocytes [8]. The role of liver fibrosis in the pathogenesis of HCC has been clearly identified, but the effect on prognosis has not yet reached an identical conclusion. A study including 189 HCC patients demonstrated that progressive fibrosis had no impact on outcome until cirrhosis was reached and only cirrhosis affected overall survival (OS) and recurrence-free survival [9]. However, Hung et al. found that minimal fibrosis was related with better survival and lower recurrence incidence by analyzing 76 HCC patients [10]. It seems controversial about the effect of liver fibrosis on HCC prognosis. Therefore, we expect to explore the impact of liver fibrosis on HCC prognosis by extracting a large amount of cases from SEER research database.

## Methods

### Data source

This study was performed using data from the SEER Program (http://www.seer.cancer.gov) SEER*Stat Database (Version 8.3.4). The SEER program of the National Cancer Institute consists of 20 cancer registries, covering 9,675,661 cases in the United States from 1973 to 2014. The SEER database, which is published routinely, includes patients’ information on demographics, primary tumor site, tumor morphology, stage at diagnosis, first course of treatment and the follow up for survival. Therefore, the SEER database is available for cancer-based epidemiology and survival analysis.

### Study population

**Fig. 1:**
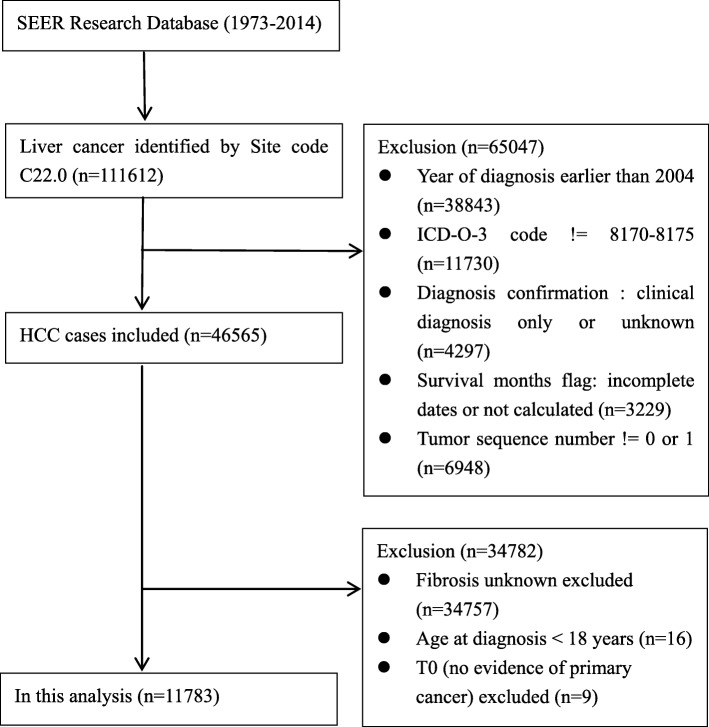
Flowchart displaying the selection procedure of HCC cases in SEER database

All patients were pathologically diagnosed as liver cancer by morphological code (C22.0) between 2004 and 2014 from SEER database. Based on International Classification of Disease for Oncology, third Edition (ICDO-3) for HCC (8170/2, 8170/3, 8171/3,8172/3, 8173/3, 8174/3, 8175/3), these patients were histologically confirmed as HCC [11]. Patients who were less than 18 years at diagnosis, had unknown survival time or diagnosed clinically only were excluded. We also excluded cases with unknown fibrosis score. Only liver cancer as primary cancer or the first cancer of multiple primary cancers was included. As shown in Fig 1, 11,783 cases matching the inclusion and exclusion criteria were finally chosen in this analysis.

### Data extraction

Demographic information (age, sex, race, and marital status), clinical characteristics (year of diagnosis, tumor size, TNM stage, SEER stage, AFP level, pathological grade, and fibrosis status) and treatment were extracted from the SEER database. Most variables, including sex, race, TNM stage, SEER stage, AFP level, pathological grade and fibrosis score used the original classification of SEER database. In SEER database, there are three categories about fibrosis score (000, 001 and 009), which means Fibrosis score 0–4 (none to moderate fibrosis), Fibrosis score 5–6 (severe fibrosis or cirrhosis) and Fibrosis score not recorded, respectively. Therefore, fbrosis score (also called Ishak score) was divided into low fibrosis group (fibrosis score 0–4) and high fibrosis group (fibrosis score 5–6). All liver fibrosis and HCC pathology are confirmed by liver biopsy. There are three ways to obtain a liver biopsy: (1) percutaneous (the most common method), (2) transjugular or transfemoral, and (3) laparoscopic. TNM stage was based on the American Joint Committee on Cancer (AJCC) Cancer Staging Manual (6th edition). In addition, we divided age into two groups at the age of 60, around the average level of this study. Due to the similar survival disadvantages of being unmarried (divorced, separated, widowed, and single), we clustered those together as the unmarried group compared with married group in further analysis. In SEER database, 6 kinds of codes are used to describe AFP level, including 000, 010, 020, 030, 080 and 999, which means test not done, positive/elevated, negative/normal, borderline, ordered, unknown or no information, respectively. Finally, cases enrolled in this study have 4 categories, positive/elevated, negative/normal, borderline, and unknown. HCC therapies were categorized into four groups: none, local tumor destruction, surgical resection and liver transplantation. As described in SEER database, tumor destruction includes photodynamic therapy, electrocautery, fulguration, cryosurgery, laser, percutaneous ethanol injection, heat-radio-frequency ablation and other local tumor destruction. Surgical resection includes wedge or segmental resection, lobectomy and extended lobectomy. Further details about the data were obtained referred to the SEER Data Management System User Manual (https://seer.cancer.gov/tools/codingmanuals/index.html).

### Statistical analyses

The categorical variables and continuous variables with different fibrosis status were compared by using the Chi-squared test and Student’s t-test, respectively. Death was treated as events. Accordingly, alive were treated as censored observation for OS, and alive or deaths from other causes were treated as censored observation for DSS. The OS was derived from the date of the diagnosis to the date of death. The primary endpoint was disease-specific survival (DSS), defined as interval from the date of the diagnosis to the date of cancer-specific death. Univariate and multivariate Cox proportional hazard models were built to determine hazard ratios (HRs) and 95% confidence intervals (CIs) for OS and DSS. Decision Curve Analysis (DCA), as a suitable method for evaluating alternative diagnostic and prognostic strategies, was also used to evaluate the effect of fibrosis on HCC prognosis. Statistical analyses were performed using the SAS 9.2 software (SAS Institute Inc., Cary, NC, USA) and R version 3.4.2 (R Development Core Team 2011). The R package “rmda” was used for the decision curve analysis. *P* value less than 0.05 with two tailed was considered to be statistically significant.

## Results

A total of 11,783 eligible patients were identified during the ten-year study period (between 2004 and 2014), including 9275 male and 2508 female patients. Of these, 2321 (19.7%) were with none to moderate fibrosis, and 9462 (80.3%) with severe fibrosis or cirrhosis. Patients in high fibrosis group were younger than those in low fibrosis group (61.0 ± 9.2 vs. 62.8 ± 11.8, *p* < 0.001), and had a greater proportion of Non-Hispanic Whites (70.9%), a lower proportion of married ones (50.9%), more frequency (80.6%) in latest years of diagnosis (2008–2014). As for tumor characteristics, low fibrosis group had more-higher frequency in large tumor size (greater than 2 cm), negative AFP value, and well or moderately differentiated pathological grade. Compared to severe fibrosis or cirrhosis cases, patients with none to moderate fibrosis had more proportions in localized and distant SEER stage, as well as TNM stage III/IV tumors. Apart from patients who had no treatment, the vast majority of low fibrosis group received surgical resection, but high fibrosis group mainly underwent tumor destruction and liver transplantation. All basic demographic and tumor characteristics between two groups were presented in Table 1.

**Table 1:** Baseline demographic and tumor characteristics of patients in SEER database

Variables		Fibrosis Score		*P* Value
		0–4	5–6	
Age, years		62.8 ± 11.8	61.0 ± 9.2	< 0.001
Age	< 60	931(40.1)	4457(47.1)	< 0.001
	≥ 60	1390(59.9)	5005(52.9)	
Sex	Male	1804(77.7)	7471(79.0)	0.194
	Female	517(22.3)	1991(21.0)	
Ethnicity	White	1352(58.2)	6709(70.9)	< 0.001
	Asian or Pacific Island	621(26.7)	1448(15.3)	
	Black	312(13.5)	1101(11.6)	
	American Indian/Alaska Native	25(1.1)	157(1.7)	
	Unknown	11(0.5)	47(0.5)	
Marital Status	Married	1298(55.9)	4818(50.9)	< 0.001
	Unmarried	932(40.2)	4303(45.5)	
	Unknown	91(3.9)	341(3.6)	
Year of Diagnosis	2004–2007	670(28.9)	1833(19.4)	< 0.001
	2008–2011	864(37.2)	3918(41.4)	
	2012–2014	787(33.9)	3711(39.2)	
TNM Stage	I	944(40.6)	3520(37.2)	< 0.001
	II	437(18.8)	2536(26.8)	
	III	524(22.6)	1840(19.4)	
	IV	280(12.1)	869(9.2)	
	Unknown	136(5.9)	697(7.4)	
SEER Stage	Localized	1365(58.8)	5366(56.7)	< 0.001
	Regional	621(26.7)	3015(31.8)	
	Distant	299(12.9)	910(9.6)	
	Unstaged	36(1.6)	171(1.8)	
Pathological Grade	Well differentiated	394(17.0)	1055(11.2)	< 0.001
	Moderately differentiated	658(28.3)	1484(15.7)	
	Poorly differentiated	279(12.0)	524(5.5)	
	Undifferentiated	22(1.0)	39(0.4)	
	Unknown	968(41.7)	6360(67.2)	
AFP	Negative	576(24.8)	2116(22.4)	< 0.001
	Positive	1324(57.1)	6234(65.9)	
	Borderline	12(0.5)	22(0.2)	
	Unknown	409(17.6)	1090(11.5)	
Tumor Size	< 2 cm	179(7.7)	1227(13.0)	< 0.001
	≥ 2 cm	1918(82.6)	7386(78.0)	
	Unknown	224(9.7)	849(9.0)	
Therapy	None	1190(51.3)	6536(69.1)	< 0.001
	Tumor Destruction	247(10.6)	1251(13.2)	
	Surgical Resection	733(31.6)	639(6.8)	
	Liver Transplantation	137(5.9)	1022(10.8)	
	Unknown	14(0.6)	14(0.1)	

A negative correlation was observed between tumor diameter and fibrosis score (*r* = − 0.16, *P* < 0.001), with smaller tumor size seen among patients with severer liver fibrosis. Meanwhile, high fibrosis score was correlated with advanced pathology grade (*r* = 0.19, *P* < 0.001). As shown in Table 2, compared with low fibrosis group, high fibrosis group was associated with poor OS (HR = 1.16, 95%CI: 1.10–1.23) and poor DSS (HR = 1.11, 95%CI: 1.05–1.19) in the univariate Cox regression models. And elder age (≥ 60 years), male, Black or American Indian/Alaska native ethnicity, unmarried status, poorly or undifferentiated pathology grade, positive AFP, large tumor size (≥ 2 cm) were regarded as risk factors for poor survival with significant difference (All *P* < 0.05). Asian or Pacific Island ethnicity was regarded as protective factor for survival (HR = 0.73, 95%CI: 0.68–0.78). Furthermore, there were obvious trends of year of diagnosis, TNM stage, SEER stage and therapy on HCC prognosis in the univariate models. The more recent the disease was diagnosed, the better the survival outcome was. With the increase of severity in TNM stage and SEER stage, the survival rate gradually decreased for both OS and DSS. Compared to none therapy, hazard ratios of overall survival were 0.40 (95%CI: 0.37–0.43) for tumor destruction, 0.29 (95%CI: 0.27–0.32) for tumor surgical resection, and 0.12 (95%CI: 0.10–0.13) for liver transplantation. The similar finding of therapy effect on DSS was also demonstrated in Table 2.

**Table 2:** Univariate Cox model analyses for overall and disease-specific survival

Variables		Overall Survival		Disease-specific Survival	
		HR (95%CI)	P Value	HR (95%CI)	*P* Value
Age	< 60	Reference		Reference	
	≥ 60	1.12(1.07–1.17)	< 0.001	1.13(1.07–1.19)	< 0.001
Sex	Male	Reference		Reference	
	Female	0.89(0.84–0.95)	< 0.001	0.89(0.84–0.95)	< 0.001
Ethnicity	White	Reference		Reference	
	Asian or Pacific Island	0.73(0.68–0.78)	< 0.001	0.73(0.68–0.78)	< 0.001
	Black	1.16(1.08–1.24)	< 0.001	1.14(1.05–1.23)	0.001
	American Indian/Alaska Native	1.23(1.03–1.46)	0.020	1.28(1.06–1.54)	0.012
	Unknown	0.59(0.39–0.88)	0.009	0.60(0.39–0.94)	0.024
Marital Status	Married	Reference		Reference	
	Unmarried	1.34(1.28–1.40)	< 0.001	1.31(1.25–1.38)	< 0.001
	Unknown	1.23(1.09–1.39)	< 0.001	1.17(1.02–1.34)	0.028
Year of Diagnosis	2004–2007	Reference		Reference	
	2008–2011	0.92(0.87–0.97)	0.002	0.94(0.88–1.00)	0.039
	2012–2014	0.85(0.79–0.90)	< 0.001	0.85(0.79–0.91)	< 0.001
TNM Stage	I	Reference		Reference	
	II	1.17(1.10–1.25)	< 0.001	1.28(1.19–1.38)	< 0.001
	III	3.24(3.04–3.45)	< 0.001	3.96(3.70–4.25)	< 0.001
	IV	6.11(5.67–6.60)	< 0.001	7.56(6.96–8.22)	< 0.001
	Unknown	3.86(3.54–4.20)	< 0.001	4.39(3.99–4.83)	< 0.001
SEER Stage	Localized	Reference		Reference	
	Regional	2.25(2.14–2.37)	< 0.001	2.56(2.42–2.71)	< 0.001
	Distant	5.30(4.95–5.69)	< 0.001	6.29(5.83–6.78)	< 0.001
	Unstaged	3.96(3.41–4.61)	< 0.001	4.06(3.42–4.81)	< 0.001
Pathological Grade	Well differentiated	Reference		Reference	
	Moderately differentiated	1.04(0.95–1.14)	0.399	1.09(0.99–1.21)	0.088
	Poorly differentiated	1.74(1.56–1.94)	< 0.001	2.00(1.78–2.26)	< 0.001
	Undifferentiated	1.65(1.22–2.23)	0.001	1.81(1.30–2.51)	< 0.001
	Unknown	1.83(1.70–1.98)	< 0.001	1.91(1.75–2.08)	< 0.001
AFP	Negative	Reference		Reference	
	Positive	1.77(1.66–1.88)	< 0.001	1.88(1.76–2.02)	< 0.001
	Borderline	1.16(0.74–1.83)	0.513	1.45(0.91–2.31)	0.119
	Unknown	1.60(1.48–1.74)	< 0.001	1.64(1.49–1.80)	< 0.001
Tumor Size	< 2 cm	Reference		Reference	
	≥ 2 cm	2.20(2.01–2.40)	< 0.001	2.69(2.41–2.99)	< 0.001
	Unknown	6.98(6.28–7.75)	< 0.001	8.94(7.90–10.12)	< 0.001
Therapy	None	Reference		Reference	
	Tumor Destruction	0.40(0.37–0.43)	< 0.001	0.36(0.33–0.40)	< 0.001
	Surgical Resection	0.29(0.27–0.32)	< 0.001	0.28(0.26–0.31)	< 0.001
	Liver Transplantation	0.12(0.10–0.13)	< 0.001	0.07(0.06–0.08)	< 0.001
	Unknown	1.34(0.91–1.99)	0.140	1.35(0.88–2.07)	0.174
Fibrosis Score	0–4	Reference		Reference	
	5–6	1.16(1.10–1.23)	< 0.001	1.11(1.05–1.19)	< 0.001

Table 3 displayed multivariate Cox proportional hazard regression models for OS and DSS. Elder age (≥60 years), unmarried status, severe TNM stage, positive AFP, large tumor size (≥ 2 cm), high fibrosis score were regarded to be significant risk factors for poor overall prognosis. Female, Asian ethnicity, latest year of diagnosis, and therapy were regarded to be significant protective factors for OS. However, Black and American Indian ethnicity and borderline AFP were not remarkably related with poor overall prognosis. Risk factors for HCC poor disease-specific prognosis were similar with those for overall prognosis except that gender was not significant protective factor for DSS. Whereas, severe fibrosis did not have a significantly impact on poor DSS (HR = 1.05, 95%CI: 0.98–1.13, *p* = 0.139) in the multivariate Cox regression model. Furthermore, regardless of TNM stage and therapy, there was no significant difference of OS and DSS between the low and high fibrosis groups (Table 4).

**Table 3:** Multivariate Cox model analyses for overall and disease-specific survival

Variables		Overall Survival		Disease-specific Survival	
		HR (95%CI)	*P* Value	HR (95%CI)	*P* Value
Age	< 60	Reference		Reference	
	≥ 60	1.12(1.07–1.18)	< 0.001	1.13(1.07–1.19)	< 0.001
Sex	Male	Reference		Reference	
	Female	0.94(0.89–0.99)	0.042	0.96(0.90–1.02)	0.184
Ethnicity	White	Reference		Reference	
	Asian or Pacific Island	0.77(0.72–0.82)	< 0.001	0.76(0.71–0.82)	< 0.001
	Black	1.04(0.97–1.12)	0.228	1.01(0.94–1.09)	0.807
	American Indian/Alaska Native	1.07(0.90–1.27)	0.464	1.11(0.92–1.34)	0.279
	Unknown	0.63(0.42–0.94)	0.022	0.67(0.43–1.04)	0.073
Marital Status	Married	Reference		Reference	
	Unmarried	1.13(1.08–1.19)	< 0.001	1.10(1.05–1.16)	< 0.001
	Unknown	1.06(0.94–1.20)	0.346	1.00(0.87–1.14)	0.941
Year of Diagnosis	2004–2007	Reference		Reference	
	2008–2011	0.80(0.76–0.85)	< 0.001	0.82(0.77–0.87)	< 0.001
	2012–2014	0.75(0.70–0.80)	< 0.001	0.76(0.70–0.82)	< 0.001
TNM Stage	I	Reference		Reference	
	II	1.14(1.07–1.22)	< 0.001	1.25(1.16–1.35)	< 0.001
	III	2.29(2.15–2.45)	< 0.001	2.70(2.51–2.90)	< 0.001
	IV	3.48(3.21–3.77)	< 0.001	4.13(3.79–4.51)	< 0.001
	Unknown	1.99(1.81–2.18)	< 0.001	2.17(1.96–2.41)	< 0.001
AFP	Negative	Reference		Reference	
	Positive	1.41(1.32–1.50)	< 0.001	1.46(1.36–1.57)	< 0.001
	Borderline	0.82(0.52–1.29)	0.385	1.01(0.63–1.61)	0.978
	Unknown	1.34(1.23–1.45)	< 0.001	1.34(1.22–1.48)	< 0.001
Tumor Size	< 2 cm	Reference		Reference	
	≥ 2 cm	1.61(1.48–1.77)	< 0.001	1.85(1.66–2.06)	< 0.001
	Unknown	2.69(2.40–3.02)	< 0.001	3.15(2.76–3.59)	< 0.001
Therapy	None	Reference		Reference	
	Tumor Destruction	0.53(0.49–0.57)	< 0.001	0.50(0.46–0.55)	< 0.001
	Surgical Resection	0.38(0.35–0.42)	< 0.001	0.37(0.33–0.41)	< 0.001
	Liver Transplantation	0.16(0.15–0.19)	< 0.001	0.10(0.08–0.12)	< 0.001
	Unknown	0.76(0.51–1.13)	0.174	0.74(0.48–1.14)	0.175
Fibrosis Score	0–4	Reference		Reference	
	5–6	1.09(1.02–1.16)	0.007	1.05(0.98–1.13)	0.139

**Table 4:** Analysis of fibrosis on OS and DSS stratified by TNM stage and therapy

TNM Stage	Therapy	Overall Survival		Disease-specific Survival	
		HR (95%CI)	P Value	HR (95%CI)	*P* Value
I + II	No	1.03(0.91–1.17)	0.612	0.98(0.85–1.12)	0.756
	Yes	1.11(0.98–1.27)	0.098	1.08(0.93–1.26)	0.302
III + IV	No	1.09(0.99–1.20)	0.085	1.07(0.97–1.19)	0.178
	Yes	1.08(0.85–1.38)	0.541	1.01(0.78–1.31)	0.956

**Fig. 2:**
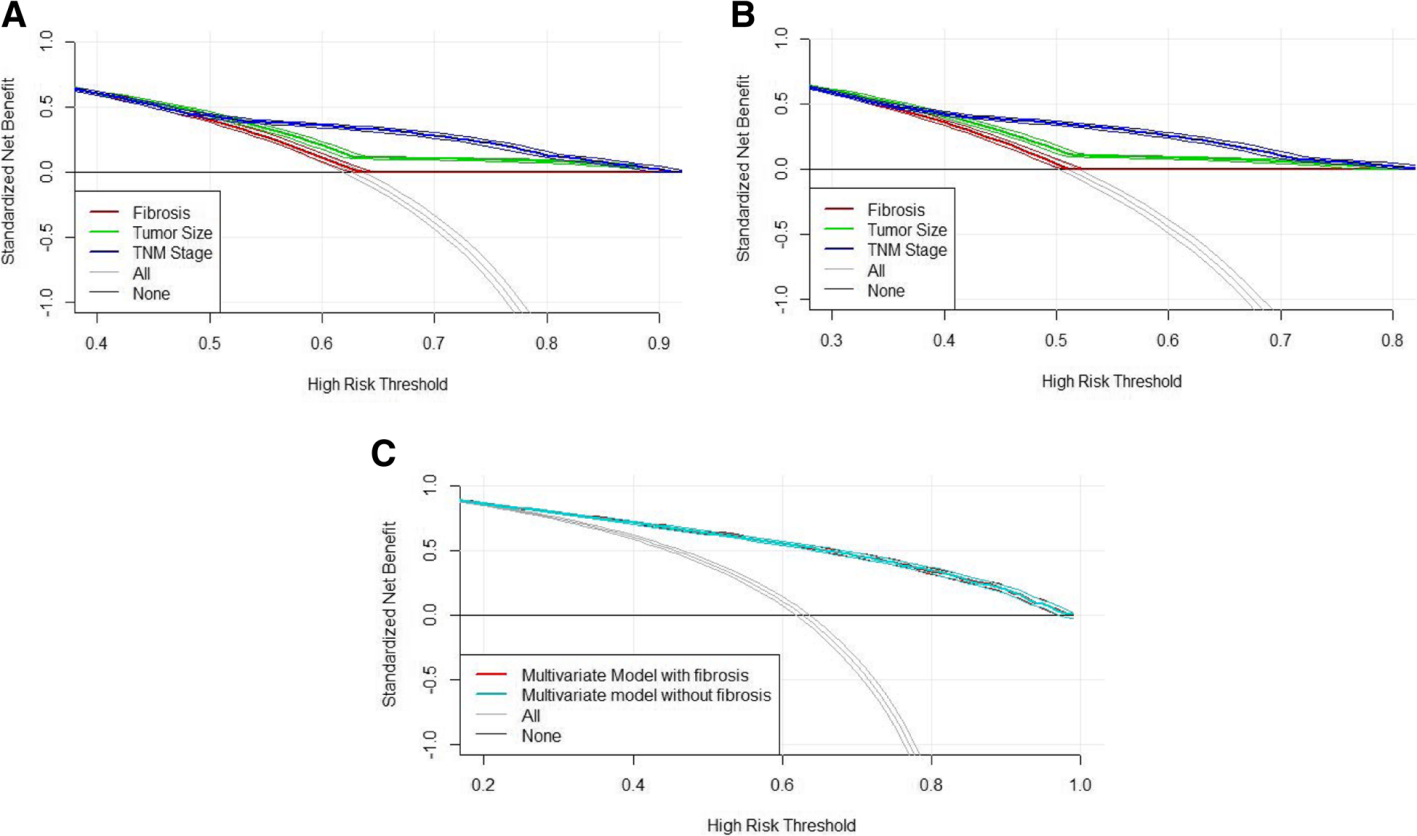
Decision curve analysis of fibrosis in patients with HCC. **a** The net benefit plotted using fibrosis, TNM stage, tumor size for overall survival. **b** The net benefit plotted using fibrosis, TNM stage, tumor size for disease-specific survival. **c** The net benefit plotted using multivariate models with and without fibrosis for overall survival

In addition, the result of DCA presented that liver fibrosis was inferior to predict OS and DSS of patients with HCC in the comparison with TNM stage and tumor size (Fig 2a and Fig 2b). The ability of overall survival prediction was similar between multivariate models with or without fibrosis (Fig 2c).

## Discussion

Our study finally enrolled 11,783 HCC cases, and found that high fibrosis score was significantly related with poor OS (HR = 1.09, 95%CI: 1.02–1.16), but not with poor DSS (HR = 1.05, 95%CI: 0.98–1.13). With the stratification of TNM stage and therapy, liver fibrosis had no significant impact both on OS and DSS. Moreover, the predictive sensitivity of fibrosis to survival outcome was lower than other clinicopathological characteristics, such as TNM stage and tumor size by DCA.

Demographic characteristics (including age, ethnicity, marital status), year of diagnosis, and clinical features (like TNM stage, SEER stage, pathological grade, AFP level, tumor size, therapy) were regarded as prognostic factors for HCC survival outcomes, which was similar with previous studies [12–15]. Some trends for HCC prognosis were found, especially in year of diagnosis, TNM stage, SEER stage and therapy. The survival outcome becomes better with time, which may be a result of advancing examination technology, such as CT and MRI, and more accurate curative treatment [3]. For therapy strategy, liver transplantation presents the best prognosis, followed by surgical resection and tumor destruction in both univariate and multivariate cox model analyses. Surgical resection, including wedge or segmental resection, lobectomy and extended lobectomy, is still the most important and routine treatment to improve the survival outcome for most HCC patients. Liver transplantation is the best option for the unresectable HCC without metastasis since it can remove the tumor completely [16]. Tumor destruction, as an assistant therapy, including various interventional therapy here, is suitable for those relatively small HCC at special location and unresectable HCC patients [17, 18].

Previous study demonstrated that development and progression of liver fibrosis were associated with hepatocyte death and a subsequent inflammatory response [19]. At the early phase, hepatic fibrosis is reversible. However, when it progresses to cirrhosis, liver failure, hepatic encephalopathy, portal hypertension and HCC will occur [20]. Furthermore, a 189 HBV-related HCC patients who had liver resection study demonstrated that only cirrhosis, rather than progressive fibrosis, had the impact on overall survival and recurrence-free survival [9]. However, a study including 76 HCC patients with small solitary HBV-related HCC who underwent resection reported that minimal fibrosis was related with better survival and lower recurrence incidence [10]. In our study, when confined patients to those who underwent surgical resection, severe liver fibrosis had a bad impact on both OS and DSS. A retrospective study revealed that fibrosis was the independent predictor of tumor recurrence among patients who undergo hepatectomy for small HCC [21]. Kadri et al found that minimal liver fibrosis had better survival outcome in the univariate analysis for HCC patients after primary surgical liver resection. In the multivariate analysis, minimal fibrosis was associated with better overall survival, but not recurrence-free survival [22]. Our finding also showed that high fibrosis score was associated with poor OS, but not DSS. The distinct conclusions of previous relevant studies maybe result from the inadequate sample size, and that only post-operative or HBV-related HCC patients were enrolled. Herein, our study included a large amount of HCC patients no matter what kinds of treatments they received.

Although a significant effect of severe liver fibrosis on poor OS other than DSS was observed, the effect of fibrosis on OS was much smaller than other clinicopathologic characteristics, such as TNM stage, tumor size based on both multivariate cox model and DCA results. There is no doubt that prognosis becomes worse with advanced TNM and large tumor size due to HCC progression.

Since treatment was also taken into consideration as one of the factors influencing the HCC prognosis [13], the analysis stratified by TNM stage and therapy was performed. There was no significant difference of OS and DSS between the low and high fibrosis groups with stratification of TNM stage and therapy. In addition, fibrosis had little influence on prognosis of HCC because there was no big difference between multivariate models with and without fibrosis. All above pointed out that although fibrosis was related with survival outcome for HCC, it has less utility in predicting the prognosis of HCC on its own.

There are some potential limitations in this study. First, SEER database only provides categorical variable of fibrosis score (0–4 vs. 5–6). If original fibrosis score information is available, we can enrich analytical contents and obtain more detailed findings of liver fibrosis. Second, in SEER database, information about comorbidities, recurrence and adjuvant chemotherapy on HCC is not open data. Besides, since SEER information is from different registers, there may be unavoidably mistakes about the accuracy of data because no specialized staff has the responsibility to check the data completely. However, SEER quality improvement methods are developed using appropriate statistical procedures that provide measures to evaluate the performance of the SEER registries. More details can be seen in SEER database website (https://seer.cancer.gov/qi/). We also have inclusion and exclusion criteria to screen the patients, Findings in this study are convincing as the US nationwide database is utilized with a large number of cases involved, and various analyses focusing on liver fibrosis are performed.

## Conclusions

In conclusion, although liver fibrosis has a relationship with survival outcome for HCC patients, it cannot be regarded as a sensitive predictor, especially in the comparison with other important clinicopathologic characteristics, such as TNM stage and tumor size.

## Abbreviations

CIs: Confidence intervals
DCA: Decision curve analysis
DSS: Disease-specific survival
HCC: Hepatocellular carcinoma
HRs: Hazzard ratios
OS: Overall survival
SEER: Surveillance, Epidemiology, and End-Results
